# Tumor-Associated CD204-Positive Macrophage Is a Prognostic Marker in Clinical Stage I Lung Adenocarcinoma

**DOI:** 10.1155/2018/8459193

**Published:** 2018-04-16

**Authors:** Yanbin Sun, Shun Xu

**Affiliations:** Department of Thoracic Surgery, The First Hospital of China Medical University, Shenyang, China

## Abstract

**Objective:**

Macrophages are the dominant leukocytes in the tumor microenvironment. Accumulating evidence revealed that CD204-positive (CD204+) tumor-associated macrophages (TAMs) are associated with the aggressive behavior of various cancers; however, the clinical, pathological, and prognostic associations of CD204+ TAMs with the subtype of lung adenocarcinoma have not been reported.

**Methods:**

Tissue microarray and immunohistochemistry were constructed from clinical stage I lung adenocarcinomas with radical surgical resection. The intratumoral density of CD204+ cells was calculated using image analysis software for analyses. Survival analyses were performed using the Kaplan-Meier method and multivariate Cox proportional hazards regression models.

**Results:**

The intratumoral density of CD204 was correlated with T stage, nodal involvement, lymphovascular invasion, and cancer relapse after the surgery, but not with age, gender, or smoking history. The density of CD204 in non-LPD was significantly higher than that in LPD. The 5-year disease-free survival (DFS) rate of CD204 high-density group was significantly worse than that of CD204 low-density group.

**Conclusions:**

The expression of CD204 in TAMs is associated with the aggressiveness of lung adenocarcinoma. Our results suggest that a specific immune microenvironment may be associated with the biological behavior of lung adenocarcinoma.

## 1. Introduction

Lung cancer is one of the most commonly diagnosed cancers [1], and the most frequent histologic type of lung cancer is adenocarcinoma [2]. Based on the histologic features, lung adenocarcinoma can be subtyped to lepidic (LPD), acinar (ACI), papillary (PAP), solid (SOL), and mucinous (MUC), and there is mounting evidence suggesting that this classification of lung adenocarcinoma can be used for prognosis [3]. With the advancement in the diagnostic techniques, more patients with lung cancer can be diagnosed at an earlier stage. However, despite the fact that surgical resection is considered the most effective therapy for patients with stage I lung adenocarcinoma, a considerable number of these patients still develop recurrence [4]. Therefore, it is important to identify the risk factors of postoperative recurrence in order to improve the outcome of patients with stage I lung adenocarcinoma.

Cancer tissue consists of not only cancer cells but also stromal cells, both of which create the tumor microenvironment. Tumor microenvironment plays important roles in the biological behaviors of cancer cells [5–10]. Macrophages comprise a dominant portion of the leukocyte population that contributes to the host's immunity [11]. Macrophages possess tumor suppressive (M1-like) and tumor-supportive (M2-like) functions [12]. Tumor-associated macrophages (TAMs) are important players in the microenvironment of most neoplastic lesions, and accumulating evidence suggests that these key inflammatory mediators are actively involved in all aspects of tumor growth and progression [13–15]. Clinical data has indicated that a high frequency of M2-polarized TAMs, characterized by M2 markers such as CD163, CD204, and CD206, is correlated with poor prognosis of multiple cancers [15–17]. Among these M2 markers, CD204, also termed scavenger receptor A (SRA) or macrophage scavenger receptor (MSR), is highly expressed in M2-like TAMs, and CD204-positive (CD204+) macrophages are associated with poor prognosis of a variety of cancers [15, 18–21]. In addition, CD204, but not CD163+, TAMs have been shown to be a more accurate prognostic factor in esophageal squamous cell carcinoma and breast cancer [19, 21].

In lung adenocarcinoma, CD204+ macrophages constitute the tumor-promoting microenvironment, and they are proposed to be an independent prognostic factor [14, 18, 22]. However, the prognostic value of CD204+ macrophages in different subtypes of stage I lung adenocarcinoma has not been well characterized. Therefore, we examined the clinicopathological and prognostic associations of tumor-infiltrating CD204+ macrophages in patients with stage I lung adenocarcinoma.

## 2. Materials and Methods

### 2.1. Patients

A total of 182 patients with stage I lung adenocarcinoma who underwent complete resection at the First Hospital of China Medical University between 2004 and 2011 were included in this study. The subtypes of the lung adenocarcinomas included lepidic (LPD, *n* = 104), acinar (ACI, *n* = 39), papillary (PAP, *n* = 14), solid (SOL, *n* = 21), and mucinous (MUC, *n* = 4) types. No patient received neoadjuvant chemotherapy. All research protocols in the present study were approved by our Institutional Review Board.

### 2.2. Histopathological Evaluation

Hematoxylin and eosin- (H&E-) stained sections of all lung adenocarcinomas were reviewed by a pathologist blinded to the clinical outcomes. Histologic type was determined according to the World Health Organization classification [3]. All tumors were histologically diagnosed as lung adenocarcinoma and were staged according to the AJCC TNM classification system (8th edition).

### 2.3. Tissue Microarray

All the tumor specimens were retrieved from the archives of the Department of Pathology at the First Hospital of China Medical University to construct tissue microarrays. Tumor samples were fixed with formalin and embedded in paraffin. Two tissue cores were punched out of each donor paraffin block. Each region in the donor paraffin block was cored with a needle of 2 mm diameter and transferred to the recipient paraffin block. Thereafter, the H&E-stained slides were reviewed.

### 2.4. Immunohistochemical Analysis

Immunohistochemical analysis of CD204 was performed using a mouse monoclonal antibody against human CD204 (clone SRA-E5, 1 : 500; Transgenic, Kumamoto, Japan) according to the standard technique for a Ventana Benchmark XT Autostainer (Ventana Medical Systems, Tucson, AZ, USA). Antigen retrieval was carried out using Cell Conditioning Solution (CC1-Tris-based EDTA buffer, PH 8.0; Ventana Medical Systems). Visualization was achieved using the I-VIEW DAB Universal Kit (Ventana Medical Systems) and hematoxylin counterstaining [20].

### 2.5. Image Analysis

Images of immunostained slides were digitized at 20x magnification by the NanoZoomer Digital Pathology System (Hamamatsu Photonics, Hamamatsu, Japan). For digital quantification, image analysis software (Tissue Studio v.3.5; Definiens AG, Munich, Germany) was used to identify CD204+ macrophages. The percentage of the areas occupied by CD204+ cells (summed area with CD204+ cells/total cancer tissue area × 100%) was calculated for each tissue microarray core.

### 2.6. Statistical Analysis

All statistical analyses were performed using SPSS 19.0 (IBM Corp., Armonk, NY). All *p* values were two-sided. Differences were considered significant at *p* < 0.05. For the categorical data, Chi-square test was performed. Kaplan-Meier survival curve and log-rank test were used to analyze the survival. To control the confounding variables, multivariate Cox proportional hazards regression models were used. The multivariate models initially included gender, age, histologic subtype, smoking, tumor side, tumor location, tumor diameter, nodal involvement, and lymphovascular invasion.

## 3. Results

### 3.1. Patient Characteristics

Among the 182 patients, there were 98 (53.8%) females and 84 (46.2%) males. The mean age was 66.7 years (34–86 years). Eighty-nine patients had a smoking history. The diameters of the tumors ranged from 6 mm to 20 mm. There were 116 patients in T1a and 66 patients in T1b, according to the 8th TNM classification. All the patients underwent lobectomy and lymph node dissection. Nodal involvement and lymphovascular invasion were found in 16 cases (8.8%) and 41 cases (22.5%), respectively. There were 21 patients suffering from recurrence and metastasis, and 19 patients died during a median 52-month follow-up period (range: 0–60 months). The rate of 5-year disease-free survival (DFS) was 88.5%, and the 5-year overall survival (OS) rate was 89.6%.

### 3.2. Density of Tumor-Infiltrating CD204+ Macrophages in Stage I Lung Adenocarcinoma

**Figure 1:**
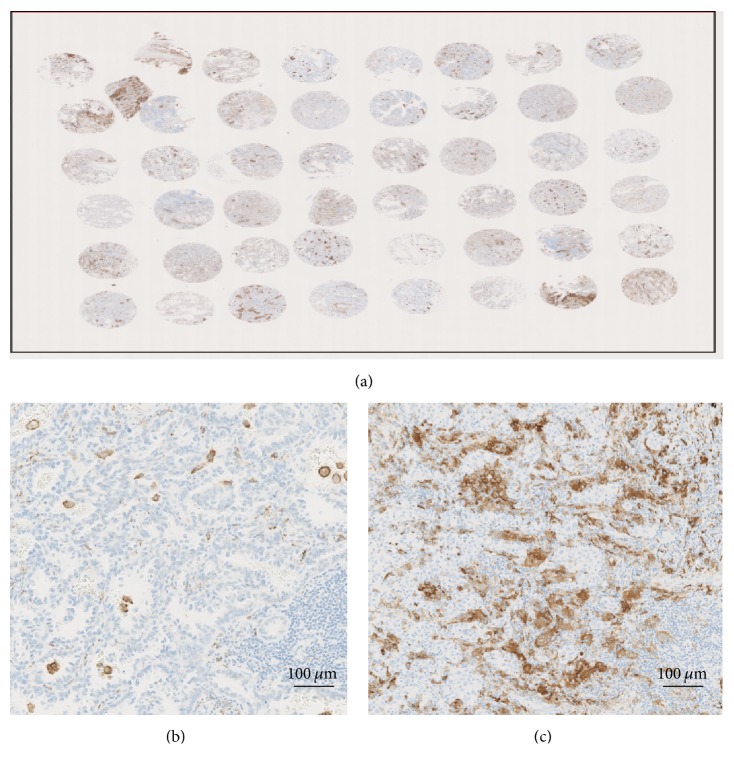
Quantitation of CD204 density in tissue microarray of stage I lung adenocarcinoma (a). Immunohistochemistry for CD204 shows low (b) and high (c) infiltration of CD204+ macrophages.

Representative photomicrographs of immunohistochemistry for CD204 are presented in Figure 1. The median density of tumor-infiltrating CD204+ cells was 0.60% (range: 0–1.12%) inside the tumors. Patients were classified into two groups based on the median density of CD204+ TAMs: a high CD204+ TAMs group and a low CD204+ TAMs group. High CD204+ density was significantly associated with the histologic subtype, T stage, nodal involvement, lymphovascular invasion, and recurrence (Table 1).

### 3.3. Tumor-Infiltrating CD204+ Macrophages in the Subtypes of Lung Adenocarcinoma

**Figure 2:**
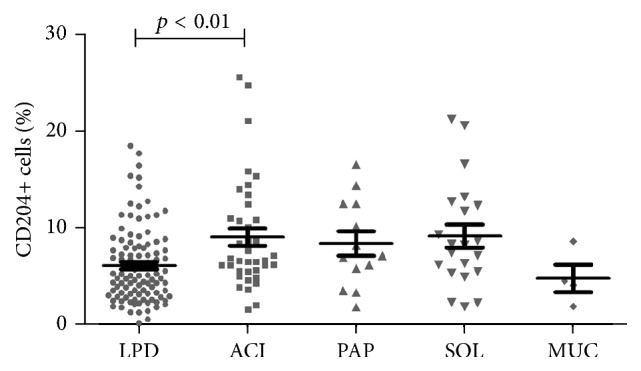
Percentages of tumor-infiltrating CD204+ macrophages in different subtypes of stage I lung adenocarcinoma.

The median density of tumor-infiltrating CD204+ cells was 0.55% (range: 0–1.12%) in LPD, 0.65% (range: 0.35–0.98%) in ACI, 0.64% (range: 0.31–0.80%) in PAP, 0.68% (range: 0–0.98%) in SOL, and 0.47% (range: 0.40–0.52%) in MUC. The density of CD204 in non-LPD (ACI, PAP, SOL, and MUC) was significantly higher than that in LPD (0.64 versus 0.55, *p* < 0.05), while there were no significant differences among ACI, PAP, and SOL (0.65 versus 0.64 versus 0.68, *p* = 0.39). In addition, the percentages of CD204+ cells in these subtypes of lung adenocarcinoma are shown in Figure 2. Compared with LPD, the percentage of CD204+ cells was significantly higher in ACI (*p* < 0.05), and the number of CD204+ cells also tended to be higher in PAP and SOL.

### 3.4. Association between CD204+ Macrophage Density and Clinical Outcomes of Lung Adenocarcinoma

**Figure 3:**
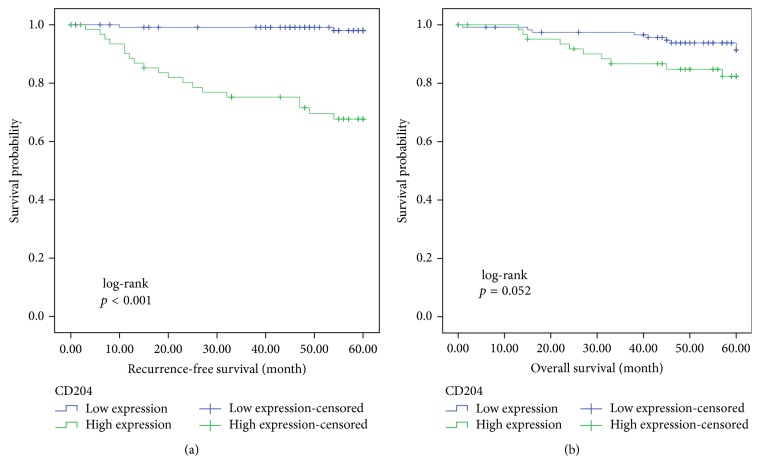
Kaplan-Meier analysis of disease-free survival (a) and overall survival (b) of patients with lung adenocarcinoma according to CD204+ macrophage density.

Kaplan-Meier analysis revealed that the 5-year DFS rate in the CD204^high^ group was significantly lower than that in the CD204^low^ group (69.8 versus 98.3%, long-rank *p* < 0.001; Figure 3(a)), but the OS rates were not significantly different between these two groups (84.1 versus 92.4%, log-rank *p* = 0.052; Figure 3(b)). High CD204 density was significantly associated with shorter DFS according to the univariate and multivariate Cox models (Table 2), but it was not significantly associated with OS in either univariate or multivariate Cox model (Table 3). The intratumoral density of CD204 was correlated with T stage (*p* < 0.05), nodal involvement (*p* < 0.001), lymphovascular invasion (*p* < 0.001), and postsurgical relapse (*p* < 0.001), but not with age (*p* = 0.745), gender (*p* = 0.981), or smoking history (*p* = 0.494). In addition, nodal involvement was significantly associated with shorter DFS and OS in univariate and multivariate analyses.

## 4. Discussion

CD204/SRA is a prototypic member of a family of transmembrane receptors termed scavenger receptors, and it is preferentially expressed in myeloid cells such as macrophages and dendritic cells [23–25]. CD204/SRA acts as a pattern recognition receptor that is capable of binding to a large variety of ligands. It is an important player in the host defense against pathogen infections [26–28] and also participates in the pathogenesis of atherosclerosis by recognizing modified lipoproteins [26, 29]. Recently, CD204+ macrophage has been identified as a crucial component of tumor-promoting microenvironment [22]. It is associated with tumor aggressiveness [18, 30] and predicts poor prognosis in a wide range of cancers [15, 18–21].

To our knowledge, the current study firstly investigated the clinicopathological and prognostic association between CD204+ TAMs and the subtypes of stage I lung adenocarcinoma according to the 8th TNM classification. Our study demonstrated that a high density of tumor-infiltrating CD204+ cells was significantly associated with a more advanced tumor stage, lymphovascular invasion, and lymph node metastasis, which are adverse prognostic factors in lung adenocarcinoma [31, 32]. Furthermore, a high density of tumor-infiltrating CD204+ cells was significantly associated with shorter DFS. However, statistical significance was not achieved for CD204 density and OS, which was probably because all the cases were in stage I. Our findings suggest that the abundance of CD204+ TAMs is a useful predictive factor for the postsurgical DFS in patients with stage I lung adenocarcinoma. It should be noted that although high CD204+ macrophage density was associated with the non-LPD histologic subtypes, the histologic subtype alone was an independent predictor of DFS. This is likely to be attributed to the differential biological behaviors of these histologic subtypes of lung adenocarcinoma.

Tumor demands nutrients, oxygen, and the ability to export metabolic wastes. These needs are addressed by tumor-associated neovascularization [33], the process of which is facilitated by macrophages [34]. Several studies have demonstrated that cancer cells in the presence of tumor-promoting TAMs exhibit enhanced invasiveness or implantation of malignant cells [35–38]. Furthermore, immunological studies have identified two different phenotypes of polarized macrophages, characterized as M1-like and M2-like macrophages [12], and the M2-like macrophages are correlated with poor prognosis of cancer [39]. A previous report proposed that CD204 could be a better marker than CD68, a pan-macrophage/monocyte marker, for the identification of tumor-promoting TAMs in patients with lung adenocarcinoma [22].

In this study, our observations suggest that tumor cells and CD204+ macrophages may cooperatively contribute to a more aggressive behavior of lung adenocarcinoma and that targeting CD204+ TAMs may be an adjuvant therapy to the conventional anticancer regimens for lung adenocarcinoma, although surgery remains the standard treatment for the patients in stage I. In addition, the diameter of the tumors included in the current study were all ≤2 cm. Whether segmentectomy could substitute lobectomy as the radical surgery for stage I lung adenocarcinoma requires further discussion and evaluation among surgeons. Based on our findings, high infiltration of CD204+ macrophages was associated with a worse outcome of stage I lung adenocarcinoma, and thus lobotomy may be a better option for this group of patients exhibiting a malignant phenotype. Our results propose a potential clinical value of a TAM marker for the assessment of surgery options, and this hypothesis needs verification in the future.

## 5. Conclusions

The present study indicates that the amount of CD204+ TAMs in stage I lung adenocarcinoma is associated with cancer aggressiveness. Our results suggest that a specific immune microenvironment may be associated with the biological behavior of lung adenocarcinoma, yet further studies are required to validate the prognostic significance of CD204+ TAMs in lung adenocarcinoma.

## Figures and Tables

**Table 1:** Correlation between CD204+ macrophage density and clinicopathological features.

Clinical or pathologic feature	Total *N*	CD204 [*n* (%)]		*p* value
		Low	High	
*All cases*	*182*	*119 (65.4)*	*63 (34.6)*	
*Gender*				0.981
Men	84	55 (65.5)	29 (34.5)	
Women	98	64 (65.3)	34 (34.7)	
*Age (years)*				0.745
<70	101	65 (64.4)	36 (35.6)	
≥70	81	54 (66.7)	27 (33.3)	
*Side*				0.769
Left	72	48 (66.7)	24 (33.3)	
Right	110	71 (64.5)	39 (35.5)	
*History of smoking*				0.494
Yes	89	56 (62.9)	33 (37.1)	
No	93	63 (67.7)	30 (32.3)	
*Histologic subtype*				0.027
Lepidic	104	75 (72.1)	29 (27.9)	
Acinar	39	23 (59.0)	16 (41.0)	
Papillary	14	5 (35.7)	9 (64.3)	
Solid	21	12 (57.1)	9 (42.9)	
Mucinous	4	4 (100)	0 (0)	
*Tumor stage*				0.008
cT1a	116	84 (71.8)	32 (28.2)	
cT1b	66	35 (53.0)	31 (47.0)	
*Nodal involvement*				<0.001
Absent	166	116 (69.9)	50 (30.1)	
Present	16	3 (18.8)	13 (81.2)	
*Lymphovascular invasion*				<0.001
Absent	141	104 (73.8)	37 (26.2)	
Present	41	15 (36.6)	26 (63.4)	
*Recurrence*				<0.001
Absent	161	117 (72.7)	44 (27.3)	
Present	21	2 (9.5)	19 (90.5)	

**Table 2:** CD204+ macrophage density in clinical stage I lung adenocarcinoma and DFS.

	Univariate analysis		Multivariate analysis	
	HR (95% CI)	*p* value	HR (95% CI)	*p* value
CD204 (high versus low)	20.91 (4.87–89.82)	<0.001	17.10 (3.37–86.68)	0.001
Gender (male versus female)	2.19 (0.91–5.29)	0.081	3.23 (0.95–10.94)	0.060
Age (≥70 versus <70 years)	1.23 (0.52–2.90)	0.633	0.60 (0.23–1.58)	0.304
Side (right versus left)	1.07 (0.44–2.58)	0.881	1.39 (0.54–3.55)	0.495
Smoking (yes versus no)	2.94 (1.14–7.57)	0.260	0.42 (0.11–1.53)	0.187
Histology (LPD versus non-LPD)	1.97 (1.47–2.64)	<0.01	2.86 (1.65–4.95)	<0.001
Tumor stage (T1a versus T1b)	3.22 (1.33–7.77)	0.009	1.92 (0.72–5.13)	0.196
Nodal involvement (yes versus no)	21.7 (9.07–51.9)	<0.001	5.64 (1.64–19.40)	0.006
LVI (yes versus no)	21.34 (7.15–63.66)	<0.001	2.48 (0.58–10.61)	0.221

The multivariate Cox regression models initially included CD204 status, gender, age, tumor side, history of smoking, histologic subtype, tumor stage, nodal involvement, and lymphovascular invasion. Backward elimination was performed with a threshold of *p* = 0.05. DFS: disease-free survival; CI: confidence interval; HR: hazard ratio; LPD: lepidic; LVI: lymphovascular invasion.

**Table 3:** CD204+ macrophage density in clinical stage I lung adenocarcinoma and overall survival.

	Univariate analysis		Multivariate analysis	
	HR (95% CI)	*p* value	HR (95% CI)	*p* value
CD204 (high versus low)	2.37 (0.96–5.85)	0.060	0.91 (0.27–3.04)	0.879
Gender (male versus female)	7.27 (2.12–24.97)	0.002	10.31 (2.47–43.01)	0.001
Age (≥70 versus <70 years)	3.03 (1.15–7.99)	0.025	3.62 (1.28–10.22)	0.015
Side (right versus left)	0.90 (0.36–2.24)	0.825	1.05 (0.41–2.65)	0.924
Smoking (yes versus no)	1.98 (0.78–5.02)	0.152	0.33 (0.09–1.17)	0.085
Histology (LPD versus non-LPD)	1.39 (0.99–1.95)	0.058	1.11 (0.67–1.84)	0.681
Tumor stage (T1a versus T1b)	2.26 (0.92–5.57)	0.076	2.15 (0.69–6.69)	0.187
Nodal involvement (yes versus no)	6.23 (2.36–16.44)	<0.001	1.55 (0.40–6.06)	0.529
LVI (yes versus no)	7.80 (3.06–19.87)	<0.001	6.81 (1.60–29.10)	0.010

The multivariate Cox regression models initially included CD204 status, gender, age, tumor side, history of smoking, histologic subtype, tumor stage, nodal involvement, and lymphovascular invasion. Backward elimination was performed with a threshold of *p* = 0.05. DFS: disease-free survival; CI: confidence interval; HR: hazard ratio; LPD: lepidic; LVI: lymphovascular invasion.
